# Plasma Seed Priming Can Improve the Early Seedling Establishment and Antioxidant Activity of Water Convolvulus Microgreens

**DOI:** 10.3390/plants14233648

**Published:** 2025-11-29

**Authors:** Mayura Veerana, Burapa Poochim, Praepun Intharasuwan, Phatlada Saphanthong, Jun-Sup Lim, Eun-Ha Choi, Gyungsoon Park

**Affiliations:** 1 Department of Applied Radiation and Isotopes, Faculty of Science, Kasetsart University, Bangkok 10900, Thailand; burapa27469@gmail.com (B.P.); praepun.in@gmail.com (P.I.); phatlada.sap@gmail.com (P.S.); 2 Plasma Bioscience Research Center, Department of Electrical and Biological Physics, Kwangwoon University, Seoul 01897, Republic of Korea; junsup117@gmail.com (J.-S.L.); ehchoi@kw.ac.kr (E.-H.C.)

**Keywords:** antioxidant activities, *Ipomoea aquatica* Forssk, microgreens, non-thermal plasma, seed germination and growth, seed priming

## Abstract

Water convolvulus (*Ipomoea aquatica* Forssk.) is a fast-growing leafy vegetable valued for its nutritional and antioxidant properties; however, suboptimal seed physiology can hinder its germination and early growth. Non-thermal plasma (NTP) treatment is an eco-friendly seed-priming method that enhances seed performance and crop quality without the use of chemical inputs. This study evaluated the effects of NTP exposure (0, 5, 10, and 20 min) using a dielectric barrier discharge (DBD) plasma with an air gas flow rate of 1.5 lpm on the germination, seedling growth, pigment and protein content, nitrogen assimilation, and antioxidant capacity of water convolvulus. Plasma treatment of seeds increased germination in a time-dependent manner. The surface hydrophilicity improved with increasing treatment time. Seedlings grown from seeds treated for 10 min exhibited longer shoots (+10.1%) and roots (+17.8%). The shoot nitrate content increased by 66.3%. At 10 min, the total phenolics and flavonoids increased by 26.5% and 37.2%, respectively, with an accompanying increase in antioxidant activity, as measured by DPPH, ABTS, and FRAP assays. These findings demonstrate that a 10 min NTP treatment of seeds improves germination, growth, nutrient assimilation, phytochemical accumulation, and antioxidant activity in water convolvulus seedlings, highlighting its potential as a sustainable and chemical-free seed-priming technology with considerable potential to enhance the productivity and nutritional quality of plant microgreens in modern agriculture.

## 1. Introduction

The increasing demand for functional foods has generated considerable interest in nutrient-dense, fast-growing crops such as microgreens. Microgreens are young seedlings of vegetables and herbs harvested within 7–21 days of germination [1,2]. They contain higher concentrations of vitamins, minerals, phenolic compounds, and antioxidants compared to those of their mature counterparts, making them an appealing alternative for health-oriented consumers and urban agriculture systems [1,2,3]. Water convolvulus (*Ipomoea aquatica* Forssk.), commonly referred to as water spinach, swamp cabbage, or kangkong, is a suitable candidate for microgreen production in various regions owing to its rapid growth, ease of cultivation, and high nutritional value. This semi-aquatic, fast-growing, leafy vegetable is cultivated and consumed across tropical and subtropical regions [4,5] with particular importance in Southeast Asia, including Thailand [6,7].

The widespread popularity of water convolvulus can be attributed to its exceptional nutritional composition, which includes high levels of vitamins such as A, C, K, and B1, essential minerals such as iron, magnesium, calcium, phosphorus, and potassium, as well as carbohydrates, proteins, and dietary fiber [8]. In addition to its basic nutritional value, it contains bioactive compounds, such as phenolics, flavonoids, and other antioxidants, which contribute to its health benefits [9]. These compounds are associated with reduced oxidative stress and prevent chronic diseases, including diabetes, cardiovascular disorders, liver injury, and certain types of cancer [9,10,11,12]. When cultivated as a microgreen, water convolvulus represents a valuable source of nutrient-dense functional foods, addressing the increasing demand for fresh produce with enhanced health benefits.

The early growth stages, particularly seed germination and seedling establishment, are crucial in determining the vigor, yield, and phytochemical profile of microgreens [1,13]. Seed dormancy, poor germination rates, and uneven growth limit uniform seed production and reduce the nutritional consistency [14]. However, successful cultivation of microgreens, including water convolvulus, requires optimized seed germination and vigorous early seedling growth to ensure high yield and quality. Therefore, enhancing the germination efficiency and early seedling growth are key targets for microgreen cultivation. Traditional seed treatments, such as priming, soaking, and chemical stimulants, have shown varying levels of effectiveness [15,16]; however, they are frequently associated with environmental constraints or food safety concerns.

Non-thermal plasma (NTP), considered an innovative green technology, has attracted considerable interest in agriculture owing to its diverse applications, such as facilitating seed germination, advancing plant growth and development, and controlling microorganisms on the surfaces of seeds and mushrooms [17,18,19,20]. Additionally, NTP can be employed to generate liquid fertilizer that enhances seed germination and seedling growth [21], as well as in hydroponic cultivation of plants [22,23,24]. NTP is a partially ionized gas comprising reactive chemical species, including reactive oxygen species (ROS), reactive nitrogen species (RNS), electrons, ions, and neutral molecules, generated at room temperature. In contrast to traditional plasma applications, which rely on high temperatures, NTP operates under ambient conditions, making it suitable for treating biological materials such as seeds, without causing thermal damage [25]. Plasma treatment has attracted considerable interest in agriculture due to its multifaceted effects on seed physiology and plant growth. Following exposure to plasma, the seeds undergo surface modifications that improve their hydrophilicity, enhance water absorption, and increase metabolic activity, thereby accelerating germination. Additionally, the reactive species generated by plasma can induce mild oxidative stress, which activates defense mechanisms and metabolic pathways in plants, leading to improved growth and development [19,26,27]. Furthermore, NTP treatment influences secondary metabolism in plants, resulting in an increased synthesis of phenolic compounds, flavonoids, and other bioactive molecules [28,29]. These compounds are crucial for plant defense against abiotic and biotic stress, as well as contributing to the nutritional and medicinal value of edible plants. Phenolics and flavonoids exhibit antioxidant properties that facilitate the scavenging of free radicals and protect cells from oxidative damage [30]. Increasing the levels of these compounds through plasma treatment represents a notable advancement in the development of functional foods with added health-promoting properties. Although several studies have reported that NTP enhances seed germination, plant growth, and the accumulation of bioactive compounds, its application to microgreens has only recently begun to receive attention. For instance, plasma treatment has been shown to stimulate growth and increase the contents of sulforaphane, glucosinolates, total phenols, and flavonoids in broccoli [31] and total phenolic content and antioxidant activity in radish microgreens [32]. However, studies focusing on water convolvulus microgreens remain scarce. Therefore, this study provides novel insights into the use of NTP seed priming for improving the germination, growth, and functional quality of water convolvulus microgreens—a crop of increasing nutritional and economic importance in Asian cuisine. Microgreens represent a unique system because of their short growth cycle, high demand for quality, and uniform production. Understanding the effects of plasma treatment on water convolvulus microgreens can provide valuable insights into the potential of NTP technology for improving the yield, nutritional quality, and antioxidant capacity of this crop.

This study aimed to investigate the effect of NTP treatment of seeds using a dielectric barrier discharge (DBD) plasma with an air gas flow rate of 1.5 lpm, on germination, seedling growth, and antioxidant capacity of water convolvulus microgreens. Specifically, this study evaluated the effects of plasma on the total phenolic content, total flavonoid content, and antioxidant activities, which are key indicators of the nutritional and functional properties of microgreens. The findings of this study are expected to contribute to the growing field of plasma agriculture by demonstrating the potential of NTP as a sustainable, cost-effective, and environmentally friendly technology for enhancing the productivity and quality of microgreens. This study provides a scientific foundation for the broader application of plasma technology in modern agricultural practices, addressing the dual challenges of food security and human health in a rapidly changing world.

## 2. Results

### 2.1. Plasma Seed Priming Enhances Germination of Water Convolvulus

**Figure 1:**
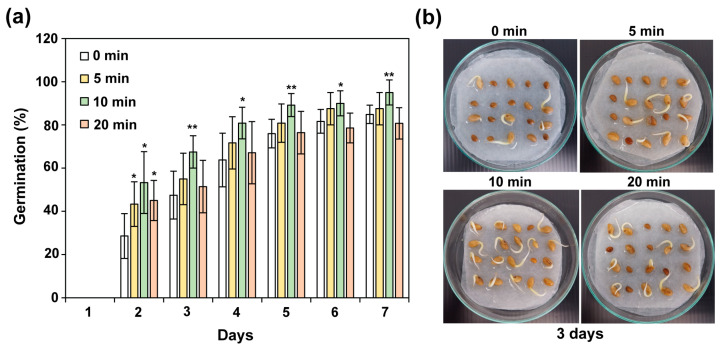
The germination percentage of water convolvulus seeds after plasma treatment on days 1–7 (**a**), and germinated seeds after plasma treatment on day 3 (**b**). Each data point represents the average of 6 replicate measurements ± standard deviation; *n* = 20 seeds per experiment. The significance of differences observed in datasets relative to the control (0 min) was tested using a Student’s *t*-test at *p* < 0.01 (**) and *p* < 0.05 (*).

The effect of non-thermal plasma (NTP) treatment on the germination of water convolvulus seeds varied with exposure duration (Figure 1a,b). After 2 days of germination, seeds exposed to NTP for 5, 10, and 20 min showed significant increases (*p* < 0.05) in germination percentage of 14.7%, 24.7%, and 16.4%, respectively, compared with untreated control seeds (0 min) (Figure 1a). Among the treatments, the 10 min exposure produced the greatest improvement, indicating that this duration was most effective for stimulating early germination. Furthermore, seeds treated for 10 min maintained significantly higher germination rates than the control throughout days 3–7 (*p* < 0.01 and *p* < 0.05), with corresponding increases of 20.0%, 17.1%, 13.2%, 8.3%, and 10.1%, respectively. These findings demonstrate that NTP exposure for 10 min optimally enhances the germination performance of water convolvulus, particularly during the early germination phase (2–3 days) (Figure 1a,b).

### 2.2. Plasma Seed Priming Increases Seed Surface Hydrophilicity

**Figure 2:**
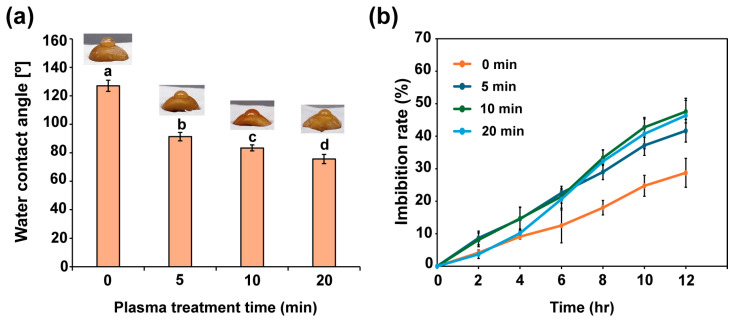
The water contact angle on the seed surface (**a**) and the imbibition rate (**b**) of the seed after plasma treatment for 0, 5, 10, and 20 min. Each value represents the mean and standard deviations of replicate measurements; *n* = 10 seeds (**a**) and *n* = 3 (**b**). The letters indicate a statistically significant difference across datasets, as determined by a one-way ANOVA followed by post hoc Tukey’s HSD test at *p* < 0.01.

The modifications to the seed surface of the water convolvulus after plasma exposure for different treatment times were investigated by measuring the hydrophilicity of the seeds, which was quantified by the angle between the seed surface and water droplets (Figure 2a). Plasma-treated seeds exhibited a decrease in the water contact angle as the irradiation time increased, compared to the untreated seeds, with average values of 127.0°, 91.3°, 83.4°, and 75.6° after plasma treatment for 0, 5, 10, and 20 min, respectively (Figure 2a). The reduction in the contact angle of the water droplets on the seed surface indicates that the hydrophilicity of the seed surface increased. Additionally, we investigated the water absorption of water convolvulus seeds after plasma treatment for 0, 5, 10, and 10 min by immersing the treated seeds in distilled water for 12 h, and the weights were recorded every 2 h. Seeds treated with plasma for 5, 10, and 20 min showed an increased capacity for water absorption, approximately 1.5 to 1.7 times higher at 12 h, with the average rates of water imbibition being 41.7%, 47.6%, and 46.4% at 12 h, respectively, which exceeded the 28.7% imbibition rate of untreated seeds (Figure 2b).

### 2.3. Plasma Seed Priming Improves Seedling Growth

**Figure 3:**
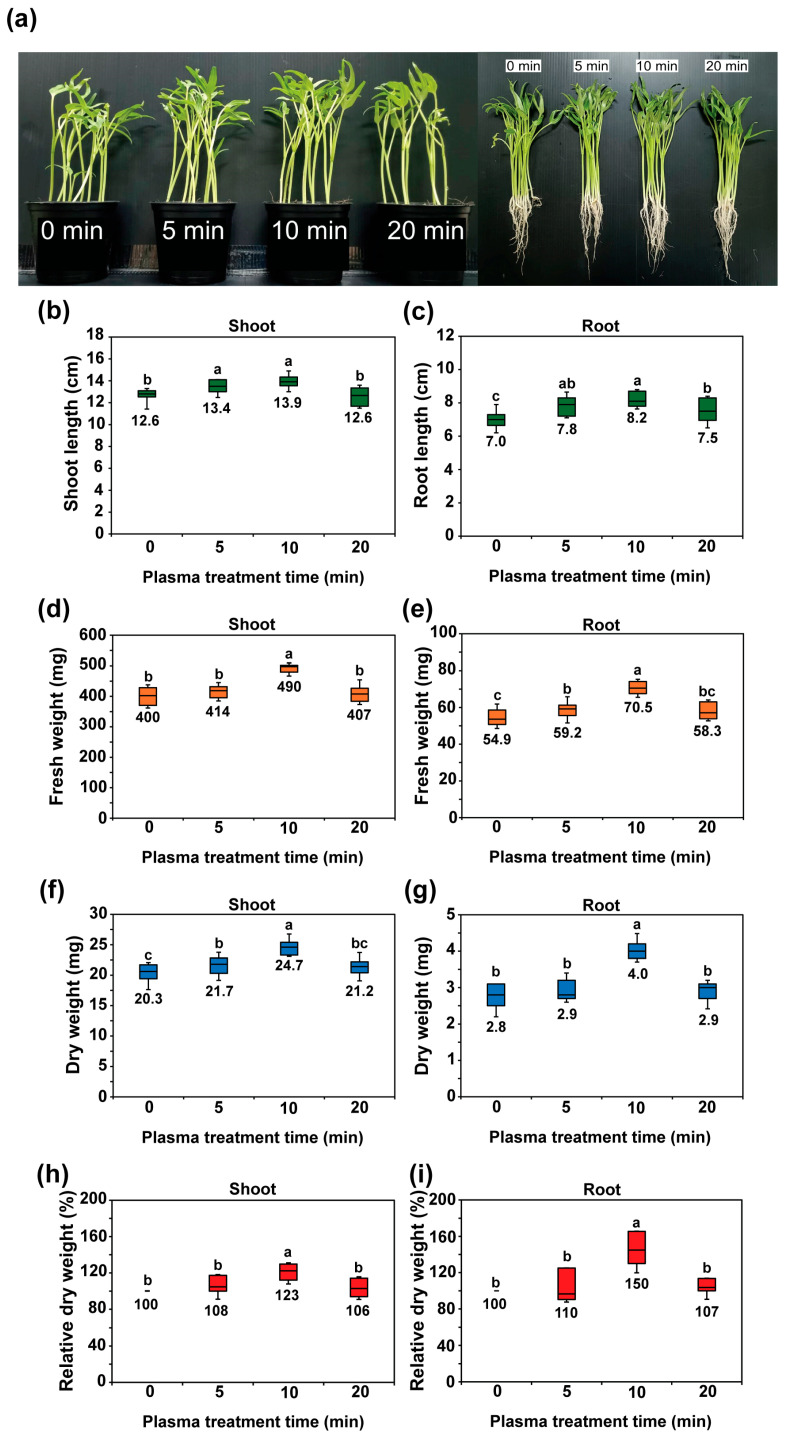
Water convolvulus plants grown in the pots and harvested at 14 days (**a**), average length of shoots (**b**) and roots (**c**), average fresh weight of shoots (**d**) and roots (**e**), average dry weight of shoots (**f**) and roots (**g**), and relative dry weight (%) of shoots (**h**) and roots (**i**) after the seeds were treated with plasma for 0, 5, 10, and 20 min on day 14 of culture. A box plot illustrates the distribution of a dataset, encompassing the minimum, first quartile (Q1), median (Q2), third quartile (Q3), and maximum; *n* = 20 plants (**b**,**c**) and *n* = 25 plants (**d**–**i**). The letters indicate a statistically significant difference across datasets, as determined by a one-way ANOVA followed by post hoc Tukey’s HSD test at *p* < 0.01.

Figure 3a shows the growth of water convolvulus seedlings cultured for 14 days after plasma treatment for 0, 5, 10, and 20 min on the seeds. Plasma-treating seeds for 5 and 10 min significantly increased shoot length by approximately 6.2% (13.4 cm) and 10.1% (13.9 cm), respectively, compared to that of the control (12.6 cm) (*p* < 0.01, Figure 3b). Additionally, plasma treatment of seeds for durations of 5, 10, and 20 min led to notably longer root lengths of approximately 12.7% (7.8 cm), 17.8% (8.2 cm), and 8.1% (8.5 cm), respectively, compared to those of the untreated seeds (7.0 cm) (*p* < 0.01, Figure 3c). Additionally, the average fresh weight (FW) of the shoots exhibited a significant increase (*p* < 0.01) after plasma treatment of the seeds for 10 min, showing an enhancement of 22.6% compared to plants from untreated seeds (Figure 3d). In contrast, both 5 and 10 min of plasma treatment notably improved the FW of roots, with increases of approximately 7.7% and 28.4%, respectively, compared to the control (*p* < 0.01, Figure 3e). The dry weight (DW) of the shoots also significantly increased after plasma treatment for 10 min by approximately 23%, compared to the control (0 min) (Figure 3f,h), whereas the DW of the roots was enhanced after plasma treatment for 10 min by 50% compared to the control (0 min) (Figure 3g,i). The findings evidently show that 10 min of plasma treatment of seeds resulted in better seedling growth, with the most notable increases observed in shoot and root lengths, as well as in both fresh and dry weights.

### 2.4. Plasma Seed Priming Increases Chlorophyll and Protein Contents in Seedlings

**Figure 4:**
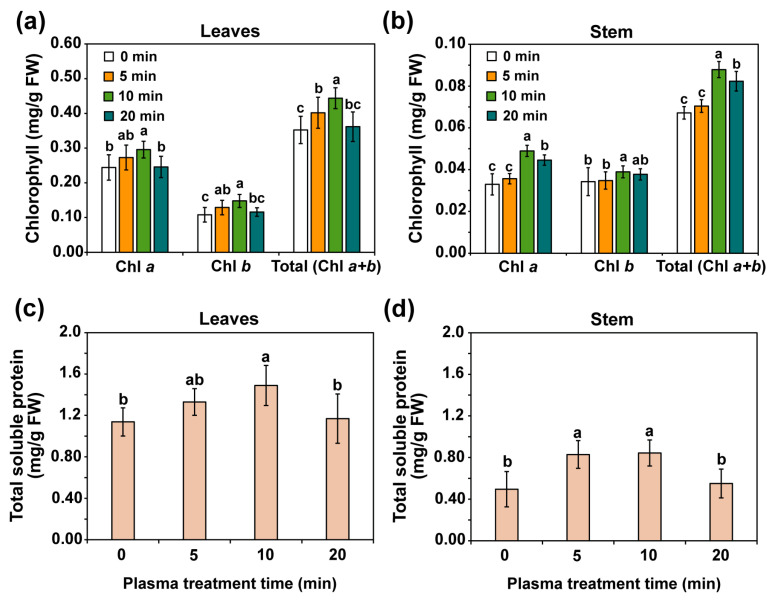
Average chlorophyll content in leaves (**a**) and stems (**b**), and total soluble protein concentration in leaves (**c**) and stems (**d**), in 14-day-old water convolvulus plants after the seeds were treated with plasma for 0, 5, 10, and 20 min. Each value represents the mean and standard deviation of replicate measurements; *n* = 9–12. The letters indicate a statistically significant difference across datasets, as determined by a one-way ANOVA followed by post hoc Tukey’s HSD test at *p* < 0.01.

The chlorophyll and total soluble protein contents in fresh leaves and stems of water convolvulus seedlings after the seeds were treated with plasma for 0, 5, 10, and 20 min and cultivated for 14 days are shown in Figure 4. The levels of chlorophyll a, chlorophyll b, and total chlorophyll per gram of fresh weight (FW) were significantly increased (*p* < 0.01) in the leaves of water convolvulus seedlings when the seeds were treated with plasma for 5 and 10 min compared with the control (0 min). However, plasma treatment for 20 min did not result in a significant increase in the levels of chlorophyll a, chlorophyll b, or total chlorophyll compared to the control. The total chlorophyll values in water convolvulus seedlings leaves that underwent plasma treatment for 0, 5, 10, and 20 min were 0.35, 0.40, 0.44, and 0.36 mg/g FW, respectively (Figure 4a). Increases of approximately 14.0% and 25.9% were observed in the total chlorophyll content of water convolvulus seedling leaves treated with plasma for 5 and 10 min, respectively, relative to that of the control (Figure 4a). In addition, plasma treatment for 10 min significantly increased the levels of chlorophyll a, chlorophyll b, and total chlorophyll in the stems. In contrast, a 20 min plasma treatment resulted in a significant increase (*p* < 0.01) in the levels of chlorophyll a and total chlorophyll in the stems compared to those of the untreated samples (Figure 4b). The total chlorophyll values in the stems of water convolvulus seedlings were 0.07, 0.07, 0.09, and 0.08 mg/g FW after plasma treatment for 0, 5, 10, and 20 min, respectively, which corresponded to increases of approximately 30.8% and 22.5% for 10 and 20 min of plasma treatment, respectively, showing no significant difference after 5 min of plasma treatment compared to those of the control (Figure 4b).

The total soluble protein content in the leaves and stems of water convolvulus seedlings was measured 14 days after the seeds were treated with plasma at different treatment times. Seedlings grown from treated seeds with plasma for 10 min showed a significant increase in total soluble protein content in leaves, with a 30.8% increase compared with that of seedlings grown from untreated seeds (*p* < 0.01, Figure 4c). Seedlings grown from treated seeds with plasma for 5 and 10 min showed a significant increase in total soluble protein content in stems, with increases of 67.2% and 70.1%, respectively, compared with the seedlings grown from untreated seeds (*p* < 0.01 (Figure 4d). However, the total soluble protein content was higher in leaves than in stems. This suggests that plasma treatment for 5 and 10 min can improve the total soluble protein content in water convolvulus seedlings.

### 2.5. Plasma Seed Priming Enhances Nitrogen Uptake in Seedlings

**Figure 5:**
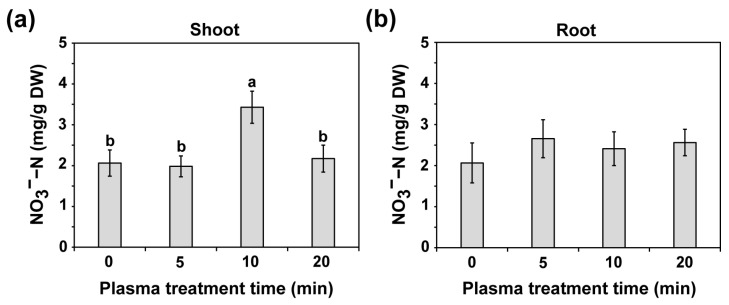
The NO_3_^−^−N levels in shoots (**a**) and roots (**b**) in 14-day-old water convolvulus plants after the seeds were treated with plasma for 0, 5, 10, and 20 min. Each value represents the mean and standard deviation of replicate measurements; *n* = 9. The letters indicate a statistically significant difference across datasets, as determined by a one-way ANOVA followed by post hoc Tukey’s HSD test at *p* < 0.01.

The NO_3_^−^−N and NH_4_^+^ concentrations were measured in the shoots and roots of water convolvulus seedlings 14 d after the plasma treatment of seeds at different treatment times (Figure 5 and Supplementary Figure S1). Regarding NO_3_^−^−N accumulation, the shoots of water convolvulus seedlings exhibited approximately 2.0, 2.0, 3.4, and 2.2 mg/g DW following plasma treatment of the seeds for 0, 5, 10, and 20 min, respectively (Figure 5a). Notably, a significant increase of approximately 66.3% was observed in the NO_3_^−^−N concentrations in the shoots of seedlings after the seeds were treated with plasma for 10 min compared with those of the untreated seeds (*p* < 0.01, Figure 5a). However, no significant differences were observed in NO_3_^−^−N concentrations in the roots (approximately 2.1–2.7 mg/g DW, Figure 5b). The NH_4_^+^ concentrations were not significantly different in both the shoots and roots compared with those of the untreated samples, which exhibited NH_4_^+^ levels of approximately 1.2–1.3 mg/g DW in the shoots (Supplementary Figure S1a) and 0.86–0.93 mg/g DW in the roots (Supplementary Figure S1b). These results indicate that plasma treatment of seeds for 10 min can increase NO_3_^−^−N accumulation in the shoots of water convolvulus seedlings.

### 2.6. Plasma Seed Priming Enhances Phenolic, Flavonoid, and Antioxidant Levels in Seedlings

**Figure 6:**
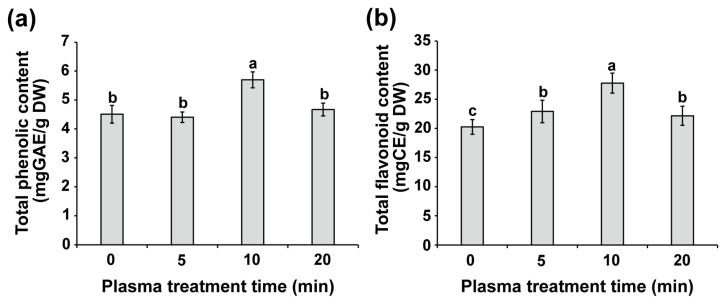
The total phenolic (**a**) and flavonoid (**b**) contents in shoots of 14-day-old water convolvulus plants after the seeds were treated with plasma for 0, 5, 10, and 20 min. Each value represents the mean and standard deviation of replicate measurements; *n* = 9–12. The letters indicate a statistically significant difference across datasets, as determined by a one-way ANOVA followed by post hoc Tukey’s HSD test at *p* < 0.01.

The total phenolic and flavonoid contents were quantified in the shoots of water convolvulus seedlings 14 days after plasma treatment of seeds for 0, 5, 10, and 20 min (Figure 6). Plasma treatment for 10 min resulted in a significant increase (*p* < 0.01) in the total phenolic content within the shoots, measuring approximately 5.7 mg GAE/g DW, which was 26.5% higher than that of the control, which had a total phenolic content of 4.5 mg GAE/g DW in the shoots (Figure 6a). Additionally, the total flavonoid contents in the shoots of water convolvulus seedlings were 20.2, 22.9, 27.8, and 22.2 mg CE/g DW following plasma treatment durations of 0, 5, 10, and 20 min, respectively. These values indicated a significant increase (*p* < 0.01) of approximately 13.1%, 37.2%, and 9.4% for plasma treatments of 5, 10, and 20 min, respectively, compared with that of the control, exhibiting the highest increase after 10 min of plasma treatment (Figure 6b). These findings indicate that treating seeds with plasma can increase the total phenolic and flavonoid levels in the shoots of water convolvulus seedlings.

**Figure 7:**
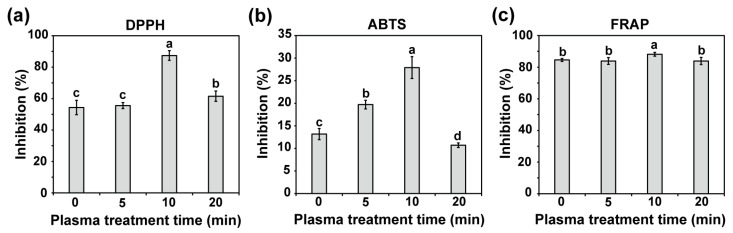
The antioxidant activity in shoots of 14-day-old water convolvulus plants after the seeds were treated with plasma for 0, 5, 10, and 20 min, measured by DPPH (**a**), ABTS (**b**), and FRAP (**c**) methods. Each value represents the mean and standard deviation of replicate measurements; *n* = 9. The letters indicate a statistically significant difference across datasets, as determined by a one-way ANOVA followed by post hoc Tukey’s HSD test at *p* < 0.01.

Antioxidant activities were measured in the shoots of water convolvulus seedlings 14 days after plasma treatment of seeds for 0, 5, 10, and 20 min using DPPH, ABTS, and FRAP assays (Figure 7 and Supplementary Table S1). A 10 min plasma treatment resulted in a significant increase (*p* < 0.01) in the percentage of inhibition of DPPH radicals (87.7%, 11.2 ± 0.53 mg TE/g DW), as measured by the DPPH assay, which was higher than that of the control (54.3%, 8.4 ± 0.48 mg TE/g DW) (Figure 7a and Supplementary Table S1). This observation was consistent with the ABTS assay, in which the percentage of inhibition was significantly increased after 5 and 10 min of plasma exposure, with values of 19.7% (10.0 ± 0.39 mg TE/g DW) and 27.9% (13.3 ± 1.0 mg TE/g DW), respectively, both exceeding that of the control (13.2%, 7.3 ± 0.51 mg TE/g DW) (*p* < 0.01, Figure 7b and Supplementary Table S1). Additionally, the FRAP assay indicated a slightly higher percentage of inhibition after 10 min of plasma exposure (88.2%, 39.1 ± 4.9 mg TE/g DW) compared with the control (84.6%, 28.6 ± 2.2 mg TE/g DW) (Figure 7c and Supplementary Table S1).

The antioxidant activities, as measured using the percentage of inhibition, were consistent with those evaluated against standards using the DPPH, ABTS, and FRAP methods, as presented in Supplementary Table S1. The Trolox content increased by 11.2 ± 0.53 mg TE/g DW in the shoots following 10 min of plasma exposure, which was significantly greater (*p* < 0.01) than that of the control (8.4 ± 0.48 mg TE/g DW), as measured by the DPPH method. When assessed using the ABTS method, the Trolox amount increased significantly at 5 and 10 min of plasma exposure to 10.0 ± 0.39 and 13.3 ± 1.00 mg TE/g DW, respectively, exceeding the control (7.3 ± 0.51 mg TE/g DW) (*p* < 0.01). Additionally, a 10 min plasma exposure increased the reducing capacity (Fe^3+^ to Fe^2+^) of the extracts, indicating an FeSO_4_ content of 39.1 ± 4.9 mg FeSO_4_/g DW, which was significantly greater (*p* < 0.01) than that of the control (28.6 ± 2.2 mg FeSO_4_/g DW, Supplementary Table S1). These results indicate that plasma treatment of seeds can potentially increase the antioxidant activity in the shoots of water convolvulus seedlings.

## 3. Discussion

This study demonstrated that seed priming with NTP significantly increased the germination of water convolvulus (*Ipomoea aquatica* Forssk.). The improvement in seed germination, particularly during the early phases (2–3 days) following plasma treatment, is consistent with previous findings in several crops, including soybean [33], wheat [34,35], tomato [36], black gram [37] and maize [38], where short-duration plasma exposure induced early germination responses. The observed improvement in the germination rate, particularly after 10 min of plasma treatment, may be attributed to multiple interrelated mechanisms. One of the primary mechanisms involves physical etching, which introduces polar functional groups and microstructural modifications of the seed coat. Plasma exposure can induce surface roughening and create micropores, as indicated by the reduced water contact angle, which increases hydrophilicity and facilitates water imbibition, thereby promoting water penetration and gas exchange, key prerequisites for initiating metabolic activities during imbibition [27,39]. These structural changes on the seed surface reduce physical dormancy and improve the capacity of seeds to rapidly absorb water, as supported by the significant increase in germination on day 2 post-treatment. Additionally, we observed that water absorption in plasma-treated seeds increased 1.66-fold compared with that in untreated seeds. Increased water uptake is critical for activating the enzymes involved in reserve mobilization during germination [40].

In addition to physical effects, NTP also produces various reactive oxygen species (ROS) and reactive nitrogen species (RNS), such as hydrogen peroxide (H_2_O_2_), ozone (O_3_), nitric oxide (NO), and others, which function as signaling molecules in seed physiology [41,42]. Moderate levels of ROS and RNS facilitate the loosening of the cell wall during radicle protrusion as well as act as secondary messengers that activate antioxidant and germination-related enzymes, including catalase, superoxide dismutase (SOD), and α-amylase [42,43]. Moreover, ROS and RNS produced during plasma exposure may act as signaling molecules that modulate gene expression and trigger the pathways involved in germination [41]. This biochemical stimulation may account for the superior germination performance observed in the 10 min plasma-treated seeds between days 2 and 7, as these seeds may have experienced a favorable ROS balance that initiated early metabolic activation without causing oxidative damage. Notably, although both the 5 and 20 min plasma treatments also increased germination rates on day 2, their effects were less consistent in the later stages compared with those of the 10 min treatment. The slightly reduced efficacy of the 20 min treatment might indicate a threshold beyond which plasma exposure becomes suboptimal or potentially detrimental. Prolonged plasma exposure can lead to oxidative stress, which impairs cellular structures or enzymes if the ROS levels exceed the antioxidant capacity of the seed tissues [44,45]. This biphasic dose–response behavior, commonly referred to as hormesis, is frequently observed in plasma seed treatment studies, where moderate doses stimulate growth, while excessive doses inhibit it [46].

Corresponding to improved germination, seedlings developed from plasma-treated seeds exhibited significantly increased growth, as evidenced by improvements in the shoot length, root length, and fresh and dry biomass. The most pronounced improvements were observed following 10 min plasma exposure, suggesting an optimal treatment duration that effectively stimulated early plant development without introducing detrimental stress. The 10 min plasma treatment produced the greatest increase in fresh and dry biomass, indicating increased water content as well as true cellular proliferation and tissue expansion. Several studies have reported similar enhancements in seedling growth parameters following plasma exposure. For example, plasma treatment of soybean (*Glycine max* L.) [33], wheat (*Triticum* spp.) [34], tomato (*Solanum lycopersicum*) [47], cumin (*Cuminum cyminum* L.) [48], and radish (*Raphanus sativus*) [49] seeds led to increased root and shoot lengths and biomass due to improved metabolic activity and water imbibition. The observed promotion of root growth is particularly relevant because root systems play a central role in nutrient uptake and plant anchorage. The roots generated from the plasma-treated seeds may develop increased lateral branching, possibly mediated by increased ROS levels and auxin sensitivity [50]. However, prolonged plasma exposure (20 min) can lead to accumulation of excessive reactive species, causing oxidative damage to cellular membranes, proteins, or DNA, thereby impairing growth rather than promoting it [51].

Furthermore, our study demonstrated that NTP seed treatment notably increased the chlorophyll and total soluble protein content in water convolvulus seedlings, with the most pronounced effects observed at treatment durations of 5 and 10 min. The increase in chlorophyll a, chlorophyll b, and total chlorophyll per gram of fresh weight (FW) in the leaves and stems indicates that plasma treatment at optimal exposure times can stimulate the photosynthetic apparatus, potentially improving the plant’s capacity to harvest light and produce biomass. These findings suggest that NTP may facilitate both chlorophyll biosynthesis and chloroplast development, consistent with studies on wheat [34,52], black gram [37], and peanut [53], where plasma exposure increased the pigment content and photosynthetic efficiency. One possible mechanism underlying the observed increase in chlorophyll content is the activation of key enzymes in the tetrapyrrole biosynthetic pathway, such as glutamyl-tRNA reductase and magnesium chelatase, which are responsible for chlorophyll biosynthesis [54]. Reactive oxygen species (ROS) and reactive nitrogen species (RNS) produced by NTP can act as signaling molecules at low concentrations, stimulating gene expression and enzymatic activity related to pigment synthesis [26,55]. The observed reduction in chlorophyll content after prolonged exposure (20 min) indicates that excessive plasma treatment may lead to mild oxidative stress or pigment degradation. At higher concentrations, ROS may damage the chloroplast membranes, photosystem complexes, and chlorophyll molecules themselves [56], thereby counteracting the positive effects of plasma-induced stimulation. This is consistent with the hormesis model in which moderate stress promotes beneficial physiological responses, whereas excessive stress is detrimental [46]. Similar time-dependent effects have been reported for cumin [57], where overexposure to plasma reduced the pigment content owing to oxidative damage.

An increase in total soluble protein content, particularly in the leaves and stems, after 10 min of plasma treatment indicates that NTP may influence primary metabolism and growth-related processes. Soluble proteins in plants include enzymes, structural proteins, and regulatory molecules that are essential for processes such as photosynthesis, respiration, and cell division [58]. Plasma-generated ROS/RNS may act as secondary messengers and upregulate the expression of genes encoding ribulose-1,5-bisphosphate carboxylase/oxygenase (RuBisCO), oxygen-evolving complex proteins, and other photosystem-associated proteins, thereby increasing protein accumulation [59]. Additionally, increased nutrient uptake following plasma treatment, due to increased root surface area and activity, provided more nitrogen, a key element for both chlorophyll and protein biosynthesis. These results are consistent with previous findings in other plant species. For example, Sadhu et al. (2017) reported that plasma treatment increased soluble protein content in mung bean sprouts [40], whereas Ling et al. (2015) observed increased soluble sugar and protein contents in oilseed rape seedlings [60].

NTP seed treatment selectively influenced nitrogen uptake and allocation in water convolvulus seedlings, particularly nitrate (NO_3_^−^−N) accumulation in shoots. Fourteen days after germination, NH_4_^+^ concentrations in both shoots and roots remained statistically unchanged across all treatment durations, indicating that plasma exposure did not significantly affect ammonium uptake or assimilation pathways under the conditions tested. In contrast, NO_3_^−^−N levels in the shoots exhibited a pronounced response to 10 min plasma treatment, whereas NO_3_^−^−N concentrations in the roots remained unaffected. This pattern indicates that NTP treatment at optimal exposure times may increase the translocation of nitrate from the roots to the shoots, rather than simply increasing root nitrate content. Nitrate transport from the roots to shoots is primarily mediated by the xylem and facilitated by nitrate transporters, such as members of the NRT1 and NRT2 families, as well as nitrate/proton symporters [61]. The observed shoot-specific nitrate increase may indicate that plasma treatment enhances the expression or activity of these transporters or increases the root-to-shoot hydraulic conductivity, thereby facilitating greater nitrate flow to aerial tissues. The unchanged NH_4_^+^ levels indicate that plasma may not have altered the activity of ammonium transporters (AMTs) or ammonium assimilation pathways, such as the glutamine synthetase/glutamate synthase (GS/GOGAT) cycle, under the given experimental conditions. However, the increased shoot NO_3_^−^−N accumulation at 10 min likely shows a more targeted plasma effect on nitrate metabolism. Nitrate serves as a nutrient as well as a signaling molecule that regulates the expression of genes related to photosynthesis, carbon metabolism, and amino acid biosynthesis [62,63]. Increased nitrate levels in shoots can stimulate the synthesis of chlorophyll and proteins, which is consistent with the significant improvements in total chlorophyll and soluble protein content observed in the same treatment group in this study. Plasma-generated reactive species, particularly nitric oxide (NO), play key roles in this process. NO interacts with nitrate signaling pathways, modulating the activity of nitrate reductase (NR) and other enzymes involved in nitrogen assimilation [64,65]. By increasing NR activity in the shoots, plasma treatment may promote more efficient nitrate reduction to ammonium, which can subsequently be incorporated into amino acids. This mechanism explains the link between the elevated shoot nitrate levels and increased protein synthesis observed after 10 min of plasma exposure.

Notably, our study demonstrated that NTP seed treatment considerably increased the accumulation of total phenolic and flavonoid compounds in the shoots of water convolvulus seedlings, particularly at an exposure time of 10 min. This observation strongly suggests that plasma exposure activates the physiological and biochemical pathways associated with secondary metabolite biosynthesis. The increased phenolic and flavonoid contents may be attributed to plasma-induced reactive oxygen and nitrogen species (RONS) produced during the treatment. RONS, including singlet oxygen (^1^O_2_), hydroxyl radicals (•OH), hydrogen peroxide (H_2_O_2_), nitric oxide (NO), and peroxynitrite (ONOO^−^), act as signaling molecules in plants, initiating defense-related gene expression and activating key enzymes in secondary metabolism [55,59]. Specifically, RONS can upregulate phenylalanine ammonia-lyase (PAL), chalcone synthase (CHS), and other key enzymes in the phenylpropanoid pathway, leading to an increased synthesis of phenolics and flavonoids [66]. Phenolic and flavonoid compounds are important for plant defense as well as exhibit considerable antioxidant capacity, which can protect plant cells from oxidative damage caused by environmental stressors [67,68]. The increase in these compounds following NTP treatment may, therefore, enhance the stress tolerance of water convolvulus seedlings, potentially improving their resilience to abiotic stresses, such as drought, salinity, and temperature extremes. Additionally, high levels of phenolics and flavonoids may enhance the nutritional and functional properties of edible shoots, thereby increasing their value as a source of natural antioxidants for human health. Similar findings have been reported for other crops. Iranbakhsh et al. (2017) found that plasma treatment significantly increased the total phenolic content in wheat seedlings [69], whereas Sirgedaite-Seziene et al. (2022) observed high flavonoid levels in Norway spruce (*Picea abies* (L.) H. Karst) after seed exposure to cold plasma [70]. Collectively, these results indicate that plasma treatment can act as a pre-sowing seed-priming strategy for improving plant phytochemical profiles. Overall, this study provides strong evidence that NTP seed treatment can be used as an effective tool to increase the production of secondary metabolites in water convolvulus seedlings, with the 10 min exposure identified as the optimal duration. The increase in phenolic and flavonoid accumulation may be attributed to plasma-generated RONS functioning as signaling molecules that activate biosynthetic pathways, with potential implications for improving both plant resilience and nutritional value. Future studies should investigate the molecular mechanisms underlying these changes, including the expression patterns of PAL, CHS, and other key enzymes, as well as the role of plasma parameters (gas type, power, and frequency) in modulating secondary metabolism.

This study evidently demonstrated that NTP seed treatment significantly enhanced the antioxidant activity of water convolvulus seedlings, with the most pronounced effects observed after 10 min of exposure. Across all three antioxidant assays (DPPH, ABTS, and FRAP), plasma treatment, particularly at the optimal duration, led to substantial increases in radical scavenging capacity and reducing power, as well as in antioxidant activity expressed in Trolox and FeSO_4_ equivalents. The observed increases in antioxidant capacity likely resulted from increased levels of phenolic and flavonoid compounds in the seedlings, as previously discussed. Phenolics and flavonoids have strong antioxidant properties, primarily owing to their ability to donate electrons or hydrogen atoms to neutralize free radicals and terminate chain reactions [71,72]. Mechanistically, the increased antioxidant activity may be attributed to the priming effect of plasma-generated reactive oxygen and nitrogen species (RONS), which act as signaling molecules to activate the plant’s antioxidant defense system. These molecules may upregulate key antioxidant enzymes, such as superoxide dismutase (SOD), catalase (CAT), and peroxidase (POD), and stimulate secondary metabolite biosynthesis via activation of the phenylpropanoid pathway [73]. This dual effect, enhancing both the enzymatic and non-enzymatic antioxidant systems, may account for the broad improvements observed in the DPPH, ABTS, and FRAP assays. From a nutritional and functional perspective, the increased antioxidant activity in water convolvulus seedlings grown from plasma-treated seeds indicates improved health-promoting properties. Diets rich in plant-derived antioxidants are associated with a reduced risk of chronic diseases, including cardiovascular disease, diabetes, and certain types of cancer [74]. Therefore, NTP seed treatment can be explored as a sustainable, chemical-free method for enhancing the nutritional quality of edible leafy vegetables, providing benefits for both agricultural productivity and human health.

## 4. Materials and Methods

### 4.1. Dielectric Barrier Discharge Plasma Source and Seed Treatment

**Figure 8:**
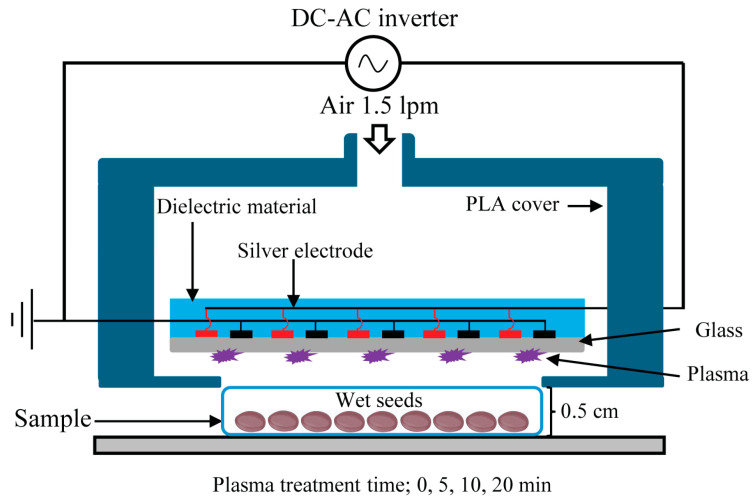
Schematic view of the dielectric barrier discharge (DBD) plasma source and seeds treatment process.

In this study, a dielectric barrier discharge (DBD) plasma source with a surface area diameter of 30 mm was used to treat water convolvulus seeds (Figure 8). Silver electrode pairs were printed onto glass substrates using a screen printer and subsequently covered with a dielectric material. Discharge occurred on the glass surface with air gas flowing at a rate of 1.5 L min^−1^ during this study. The outer cover, manufactured from polylactic acid (PLA) using a 3D printer, acted as a gas guide for the DBD surface discharge. A DC-AC inverter that produced a 42 kHz sinusoidal wave with a peak voltage of 2 kV was employed for the DBD operation. The electrical properties of DBD plasma, including the voltage and current curve, are shown in Supplementary Figure S2, and the chemical characterization of DBD plasma by Gas Fourier Transform Infrared Spectroscopy (Gas-FTIR) is shown in Supplementary Figure S3 [75].

Water convolvulus seeds were purchased from East-West Seed Co., Ltd. (Nonthaburi, Thailand). Seeds were soaked in a 2% sodium hypochlorite solution for 20 min, after which they were rinsed three times with deionized water to remove any traces of sodium hypochlorite. Following the washing procedure, the wet seeds (20 seeds) were placed at the center of a Petri dish and treated with plasma using air gas at a flow rate of 1.5 L min^−1^ for durations of 0, 5, 10, and 20 min, ensuring that the distance from the bottom of the Petri dish to the plasma surface was 0.5 cm (Figure 8).

### 4.2. Germination Efficiency and Seedling Growth Measurement

The moist seeds were subjected to air plasma treatment for 0, 5, 10, and 20 min as described above. Following treatment, germination of the seeds was evaluated by placing 20 seeds on a Petri dish covered with two layers of filter paper, and 10 mL of distilled water was added. The Petri dishes were incubated at room temperature under a photoperiod of 8 h darkness and 16 h light for 7 days. Seed germination was recorded daily, and the seed germination percentage was calculated as follows:

$$Seed\;germination\left(\%\right)=\frac{Number\;of\;germinated\;seeds}{Total\;number\;of\;seeds\;tested}\times 100$$

To evaluate the growth of the seedlings, the treated seeds were grown in plastic pots with a diameter of 7 cm and a height of 6.5 cm containing nursery soil at room temperature, maintained under a photoperiod of 8 h of darkness and 16 h of light for 14 days, and irrigated with 20 mL of deionized water every 2 days. The plants were harvested after 14 days of cultivation, then the soil was carefully removed and thoroughly washed with distilled water and dried with tissue paper. The lengths of shoots and roots were measured with a ruler, while the fresh weight (FW) of both shoots and roots was determined using a balance. Subsequently, the samples were incubated at 60 °C for 3 days, after which the dry weight was measured. The relative dry weight (%) was calculated as

$$Relative\;Dry\;weight\left(\%\right)=\frac{Dry\;weight\;of\;treated\;sample}{Dry\;weight\;of\;control\;at\;0\;min}\times 100$$

### 4.3. Measurement of Water Contact Angle and Water Imbibition on Plasma-Treated Seeds

To analyze the modifications to the seed surface after plasma exposure, we investigated the hydrophilicity of the seed surface by determining the contact angle of the water droplets. Seeds were treated with air plasma for 0, 5, 10, and 20 min, and then 2 μL of water was dropped onto the surface of the seeds. Photographs were captured immediately, and the contact angle between the seed surface and the water droplet was measured. Ten seeds were analyzed per treatment.

To assess water absorption in seeds following plasma treatment, the seeds were subjected to air plasma treatment for 0, 5, 10, or 20 min. After treatment, 10 seeds from each group were weighed (*W_0_*), then immersed in water and incubated at room temperature for 2, 4, 6, 8, 10, or 12 h. Water uptake by the treated seeds was evaluated by blotting the seeds with tissue paper to eliminate excess water, and the seeds were weighed (*W_t_*) at each incubation time point. Each treatment was performed in triplicate. The percentage imbibition rate was calculated as follows:

$$Imbibition\;rate\left(\%\right)=\frac{\left(W_{t}-W_{0}\right)}{\;W_{0}}\times 100$$

### 4.4. Measurement of Chlorophyll Content

To determine the chlorophyll content, 50 mg of fresh leaves or stems from water convolvulus seedlings cultured for 14 d were placed into a 2.0 mL tube. Subsequently, 1 mL of 95% ethanol was added to the tube, and the tubes were inverted several times to ensure thorough mixing of the samples with the ethanol solution. The tubes were then covered with aluminum foil to block light exposure and incubated at room temperature for 5 days or until all samples turned completely white. The absorbance of the extracted liquid was measured at 665 and 649 nm using a spectrophotometer (SPECTROstar^Nano^, BMG Labtech, Ortenberg, Germany). Concentrations of chlorophyll a, chlorophyll b, and total chlorophyll were calculated in mg/g of fresh weight (FW) using the following equations [76]:

Chlorophyll a (µg/mL) = 13.95 × A665 − 6.88 × A649

Chlorophyll b (µg/mL) = 24.96 × A649 − 7.32 × A665

Total chlorophyll (mg/g FW) = (Chlorophyll a + Chlorophyll b) × V/W

where A649 and A665 represent the absorption values at 649 and 665 nm, respectively, V is the volume of the extraction solution (mL), and W is the weight of fresh leaves or stems of water convolvulus seedlings (g).

### 4.5. Analysis of Total Soluble Protein Content

To quantify the total soluble protein content, 100 mg of fresh leaves or stems from water convolvulus seedlings that had been cultured for 14 days were ground in liquid nitrogen. The ground powder was then placed into 1.5 mL microtubes. Subsequently, 1 mL of 1X phosphate-buffered saline (PBS) was added to the tube, which was vortexed and then centrifuged at 12,400× *g* and 4 °C for 10 min. The supernatants were collected and transferred to new tubes. A Bradford assay kit (Bio-Rad, Hercules, CA, USA) was used to determine the concentration of total soluble proteins, as per the manufacturer’s protocol. Bovine serum albumin was used as a standard.

### 4.6. Determination of Nitrogen Uptake (NO_3_^−^−N and NH_4_^+^)

The primary nitrogen sources available for plant uptake and assimilation are NH_4_^+^ and NO_3_^−^ ions, suggesting that the intracellular concentrations of NH4^+^ and NO_3_^−^−N may be correlated with plant growth and quality. To evaluate the concentrations of NO_3_^−^−N and NH_4_^+^, fresh shoots and roots of water convolvulus seedlings grown for 14 d were collected. The shoots and roots were completely dried in an oven at 65 °C and then ground in a mortar using a pestle. To measure the NO_3_^−^−N concentration, 100 mg of the powdered shoots and roots were dissolved in 10 mL of deionized water and incubated at 45 °C for 1 h. After incubation, the suspensions were filtered through Whatman No. 40 filter paper (Whatman Inc., Maidstone, UK) and immediately analyzed for NO_3_^−^−N levels using a salicylic acid-sulfuric acid method [77], which quantifies the nitrosalicylic acid content. The filtered solution (0.1 mL) was combined with 0.4 mL of a 5% salicylic acid solution and subsequently incubated at room temperature for 20 min. Following the incubation period, 9.5 mL of 2N NaOH solution was gradually added and thoroughly mixed. The absorbance of the resulting mixture was promptly measured at 388 and 440 nm using a spectrophotometer (SPECTROstar^Nano^, BMG Labtech). A standard curve was established by reacting potassium nitrate at different concentrations (0–100 mg/L) with 5% salicylic acid and 2 N NaOH, following the previously outlined procedure. The concentration of NO_3_^−^−N in both shoots and roots was determined in micrograms per gram of dry weight.

The phenol–hypochlorite reaction was employed to assess the concentration of NH_4_^+^ [78]. A mixture was prepared by combining 10 mg of ground powder from dried shoots and roots of water convolvulus seedlings with 1 mL of deionized water and shaking for 15 min at 25 °C to facilitate the extraction of NH_4_^+^. Following extraction, the mixture was centrifuged at 12,400× *g* for 5 min, and the supernatant was carefully transferred into new tubes. To quantify the NH_4_^+^ concentration, a reaction mixture was formulated consisting of 0.1 mL of the supernatant, 0.4 mL of deionized water, 2.5 mL of phenol–sodium nitroprusside (comprising 100 mM of phenol and 0.16 mM of sodium nitroprusside), and 2.5 mL of alkaline hypochlorite (which includes 125 mM of sodium hydroxide and 5 ppm of sodium hypochlorite solution), and this was incubated at 30 °C for 10 min. After incubation, the absorbance was measured at 635 nm using a spectrophotometer (SPECTROstar^Nano^, BMG Labtech). A standard curve was created using ammonium sulfate to determine NH_4_^+^ concentration.

### 4.7. Analysis of Total Phenolic and Flavonoid Contents

#### 4.7.1. Plant Extraction

The shoots of water convolvulus seedlings cultivated for 14 days were collected. The shoots were thoroughly dried in an oven maintained at 65 °C and subsequently ground using a mortar and pestle. Dried shoots (0.5 g) were subjected to extraction with 50 mL of 80% methanol and shaken at room temperature for 48 h. Following extraction, the suspensions were filtered through a Whatman No. 1 filter paper (Whatman Inc., Maidstone, UK). The resulting extracted solution was collected to assess the total phenolic content, total flavonoid content, and antioxidant activity [79].

#### 4.7.2. Total Phenolic Content (TPC) Measurement

The total phenolic content was evaluated by combining 0.5 mL of the sample extract, 3 mL of distilled water, and 0.25 mL of Folin–Ciocalteu phenol reagent (Sigma-Aldrich, Saint Louis, MO, USA). The mixture was then incubated for 5 min. Subsequently, 1 mL of 7.5% sodium carbonate was added, and the mixture was stored in the dark for 90 min at room temperature. The absorbance of the solution was measured at 760 nm using a spectrophotometer (SPECTROstar^Nano^, BMG Labtech). The standard used to determine total phenolic content was gallic acid, and the results are reported as milligrams gallic acid equivalent per gram of dry extract (mg GAE/g DW) [79].

#### 4.7.3. Total Flavonoid Content (TFC) Measurement

The evaluation of the total flavonoid content involved a combination of the sample extract (0.5 mL) with 2 mL of distilled water and 0.15 mL of 5% sodium nitrite, followed by a 5 min incubation. After incubation, 0.15 mL of 10% aluminum chloride was added to the solution, which was then maintained in the dark for 1 min. Subsequently, 1 mL of 1 M sodium hydroxide was added to the mixture, and the volume was adjusted to 5 mL with distilled water. The absorbance was recorded at 510 nm using a spectrophotometer (SPECTROstar^Nano^, BMG Labtech). Catechin was used as the standard to generate the calibration curve. These findings are reported as catechin equivalents per gram of dry extract (mg CE/g DW) [79].

### 4.8. Determination of Antioxidant Activity

#### 4.8.1. DPPH Assay

The antioxidant activity of water convolvulus seedlings cultured for 14 days was tested using a previously reported method [80] with slight modifications. To evaluate antioxidant activity, 1 mL of the sample extract was combined with 2 mL of 0.6 mM 2,2-diphenyl-1-picrylhydrazyl (DPPH) and shaken vigorously. The control sample was also prepared by mixing 1 mL of 80% methanol with 2 mL of 0.6 mM DPPH. The mixture was incubated in the dark for 60 min at room temperature. The absorbance of the mixture was measured at 517 nm using a spectrophotometer (SPECTROstar^Nano^, BMG Labtech). The antioxidant activity of a sample was determined by the level of inhibition of DPPH radical absorption (% inhibition), which was calculated using the following equation:

$$DPPH\;inhibition\left(\%\right)=\frac{\left(A_{control}-A_{sample}\right)}{\;A_{control}}\times 100$$

where *A_control_* represents the absorbance of the DPPH solution in the absence of antioxidants, while *A_sample_* represents the absorbance of the sample. The results are expressed as mg Trolox equivalent per gram of sample based on dry weight (mg TE/g DW). A standard curve was generated using Trolox dissolved in 80% methanol.

#### 4.8.2. ABTS Assay

The antioxidant properties of the water convolvulus seedling extracts were assessed using the ABTS method, as previously described [81], with slight modifications. The radical ABTS^•+^ was generated by oxidizing ABTS (2,2′-azino-bis [3-ethylbenzothiazoline-6-sulfonic acid]) with potassium persulfate. The radical ABTS^•+^ was prepared by mixing 1:1 (*v*/*v*) of 7 mM ABTS and 4.95 mM potassium persulfate and then maintaining it at room temperature in the dark for 16 h. Subsequently, the mixture was diluted with methanol to achieve absorbance values between 1 and 1.5 at 734 nm. To assess the antioxidant activities of the extracts, each extract (0.1 mL) was combined with 3.9 mL of the ABTS^•+^ dilution and allowed to react for 30 min in the dark. A control was prepared by mixing 80% methanol and ABTS^•+^. The reduction in absorbance was recorded at 734 nm using a spectrophotometer (SPECTROstar^Nano^, BMG Labtech). The antioxidant activity of each sample was evaluated based on the percentage of ABTS^•+^ radical scavenging activity (% inhibition) using the following equation:

$$ABTS\;inhibition\left(\%\right)=\frac{\left(A_{control}-A_{sample}\right)}{\;A_{control}}\times 100$$

where *A_control_* is the absorbance of the ABTS^•+^ solution without antioxidants, and *A_sample_* refers to the absorbance of the sample after a reaction time of 30 min. Additionally, a standard curve of Trolox dissolved in 80% methanol was prepared. The results are reported as mg Trolox equivalent per gram of sample based on dry weight (mg TE/g DW).

#### 4.8.3. FRAP Assay

The reducing capabilities of the extracts, which demonstrated their antioxidant activities, were evaluated using a modified Fe^3+^ to Fe^2+^ reduction assay. The FRAP assay was performed according to a previously described method [81]. The FRAP reagent was prepared by mixing a 10:1:1 (*v*/*v*) solution of 300 mM sodium acetate solution (pH 3.6), 10 mM TPZT (2,4,6-Tris(2-pyridyl)-s-triazine), and 20 mM FeCl_3_ hexahydrate, and the reagent was dissolved homogeneously. To evaluate the antioxidant activity of the extract sample, 0.2 mL of each extract was mixed with 3.8 mL of the FRAP reagent and incubated at 37 °C for 30 min. The increase in absorbance was recorded at 593 nm using a spectrophotometer (SPECTROstar^Nano^, BMG Labtech). The control was prepared by substituting the same volume of the extract with 80% methanol. The percentage of attenuation rate (% inhibition) was calculated using the following formula [82]:

$$FRAP\;inhibition\left(\%\right)=\frac{\left(A_{sample}-A_{control}\right)}{\;A_{sample}}\times 100$$

where *A_sample_* is the absorbance of the extract sample following a reaction period of 30 min, and *A_control_* represents the absorbance of the FRAP reagent in the absence of antioxidants. Furthermore, a standard curve of FeSO_4_ dissolved in 80% methanol was established. The results are reported as milligram equivalents of FeSO_4_ per gram of dry weight (mg FeSO_4_/g DW).

### 4.9. Statistical Analysis

All data are presented as the mean ± standard deviation. All experiments were repeated two or three times, with at least three replicate measurements in each experiment. The significance of differences observed in datasets relative to the control (0 min) was tested using a Student’s *t*-test at *p* < 0.01 (**) and *p* < 0.05 (*) (Figure 1a) and one-way ANOVA, followed by a post hoc Tukey’s HSD test at *p* < 0.01 using R software version 4.4.1 (Figure 2, Figure 3, Figure 4, Figure 5, Figure 6 and Figure 7 and Supplementary Table S1); the letters indicate statistically significant differences across datasets.

## 5. Conclusions

This study demonstrated that NTP seed treatment significantly improved the germination, seedling growth, physiological and biochemical traits, and antioxidant activity of water convolvulus (*Ipomoea aquatica* Forssk.). Plasma exposure, particularly for 10 min, increased the seed surface hydrophilicity and water imbibition, which facilitated faster and higher germination rates. The seedlings grown from plasma-treated seeds showed an increase in shoot and root lengths, as well as in fresh and dry biomass, chlorophyll and soluble protein levels, nitrogen assimilation, total phenolic and flavonoid content, and antioxidant activity. Our study suggests that short-duration (optimal) plasma exposure can be an effective and eco-friendly seed priming strategy to improve the early establishment and nutritional quality of microgreens, such as water convolvulus, providing potential benefits for sustainable agriculture and functional food production. However, databases for plasma priming effects should be established using various microgreen species and plasma sources, and elucidation of the underlying mechanisms is required for application in agricultural practices.

## Data Availability

The original contributions of this study are included in this article. Further inquiries can be directed to the corresponding authors.

## Supplementary Materials

The following supporting information can be downloaded at: https://www.mdpi.com/article/10.3390/plants14233648/s1, Figure S1: The NH_4_^+^ levels in shoots (a) and roots (b) in 14-day-old water convolvulus plants after the seeds were treated with plasma for 0, 5, 10, and 20 min. Each value represents the mean and standard deviation of replicate measurements; *n* = 9. The letters indicate a statistically significant difference across datasets, as determined by a one-way ANOVA followed by post hoc Tukey’s HSD test at *p* < 0.01; Figure S2: Electrical characteristics of DBD, voltage (black line), and current curve (red line) versus time; Figure S3: The chemical characteristics of DBD by Gas Fourier Transform Infrared Spectroscopy (Gas-FTIR); Table S1: The antioxidant activity in shoots of 14-day-old water convolvulus plants after the seeds were treated with plasma for 0, 5, 10, and 20 min, expressed as milligram equivalents of standard per gram dry weight.

## Abbreviations

The following abbreviations are used in this manuscript:

NTP	Non-thermal plasma
ROS	Reactive oxygen species
RNS	Reactive nitrogen species
DBD	Dielectric barrier discharge
FW	Fresh weight
DW	Dry weight
TPC	Total phenolic content
TFC	Total flavonoid content
Chl *a*	Chlorophyll a
Chl *b*	Chlorophyll b
DPPH	2,2-diphenyl-1-picrylhydrazyl
ABTS	2,2′-azino-bis (3-ethylbenzothiazoline-6-sulfonic acid)
FRAP	Ferric Reducing Antioxidant Power assay
