# Supplementary material for: Plasma Seed Priming Can Improve the Early Seedling Establishment and Antioxidant Activity of Water Convolvulus Microgreens
Source: Plants (Basel). 2025 Nov 29;14(23):3648. doi: 10.3390/plants14233648 (PMC12694209; doi:10.3390/plants14233648)
Supplement: Supplementary file 1 [file plants-14-03648-s001.zip › plants-3946815-supplementary.pdf]

Supplementary Information

Plasma Seed Priming Can Improve the Early Seedling Establishment and Antioxidant Activity of Water Convolvulus Microgreens

Mayura Veerana [1,*], Burapa Poochim [1], Praepun Intharasuwan [1], Phatlada Saphanthong [1], Jun-Sup Lim [2], Eun-Ha Choi [2] and Gyungsoon Park [2,*]

[1] Department of Applied Radiation and Isotopes, Faculty of Science, Kasetsart University, Bangkok 10900, Thailand; fscimuv@ku.ac.th (M.V.); burapa27469@gmail.com (B.P.); praepun.in@gmail.com (P.I.); phatlada.sap@gmail.com (P.S.)

[2] Plasma Bioscience Research Center, Department of Electrical and Biological Physics, Kwangwoon University, Seoul 01897, Republic of Korea; junsup117@gmail.com (J.S.L.); ehchoi@kw.ac.kr (E.H.C.); gyungp@kw.ac.kr (G.P.)

* Correspondence: fscimuv@ku.ac.th (M.V.), gyungp@kw.ac.kr (G.P.); Tel.: +66-97-985-9297 (M.V.), +82-10-5703-1569 (G.P.)

Section S1. NH_4^+ Uptake in Seedlings

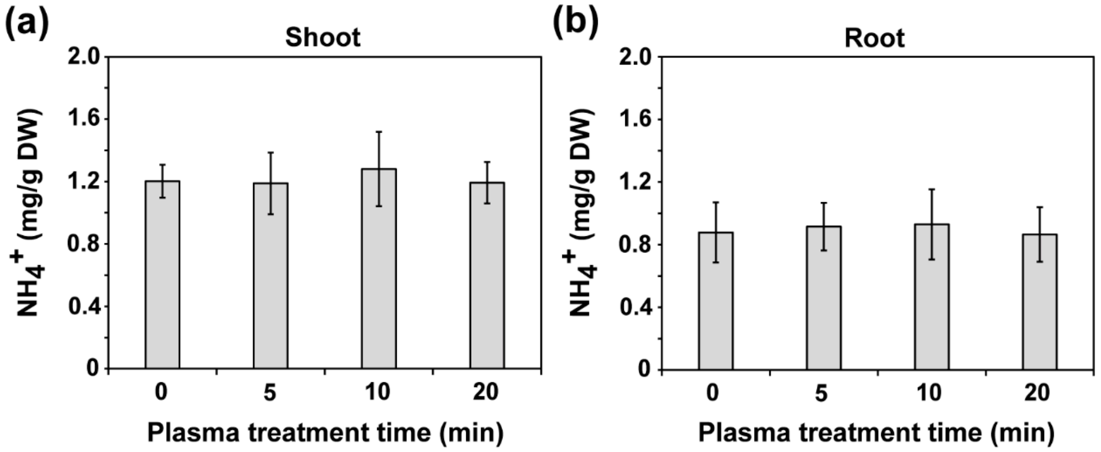

Supplementary Figure S1. The NH_4^+ levels in shoots (a) and roots (b) in 14-day-old water convolvulus plants after the seeds were treated with plasma for 0, 5, 10, and 20 min. Each value represents the mean and standard deviation of replicate measurements; *n* = 9. The letters indicate a statistically significant difference across datasets, as determined by a one-way ANOVA followed by post hoc Tukey's HSD test at $p < 0.01$.

Section S2. Plasma Characteristics

The electrical properties are shown in Supplementary Figure S2. The electrical voltage and current curves were measured using a high-voltage probe (P6015A, Tektronix Korea, Ltd., Seoul, Korea) and a current probe (P6021A, Tektronix Korea, Ltd.), respectively, and the measurements were recorded using an oscilloscope (MSOX3104T, Keysight Technologies, Santa Rosa, CA, USA). To reduce the thermal damage to the DBD, a 15% duty cycle was used in the DC-AC inverter [76]. The current curve revealed that the peaks in the plasma discharge current were caused by discharges on the DBD surface, as shown in Supplementary Figure S2. The dissipated electrical power was determined using the following expression:

$$Dissipated\ power\ (W) = Duty\ cycle \times \frac{1}{T}\int_0^T v(t)i(t)dt$$

where T represents the period of the sinusoidal wave, and v(t) and i(t) represent the recorded voltage and current waveforms, respectively. The measured dissipation power was 0.7 W. The root-mean-square values of the voltage and current were 1.56 kV and 16.4 mA, respectively.

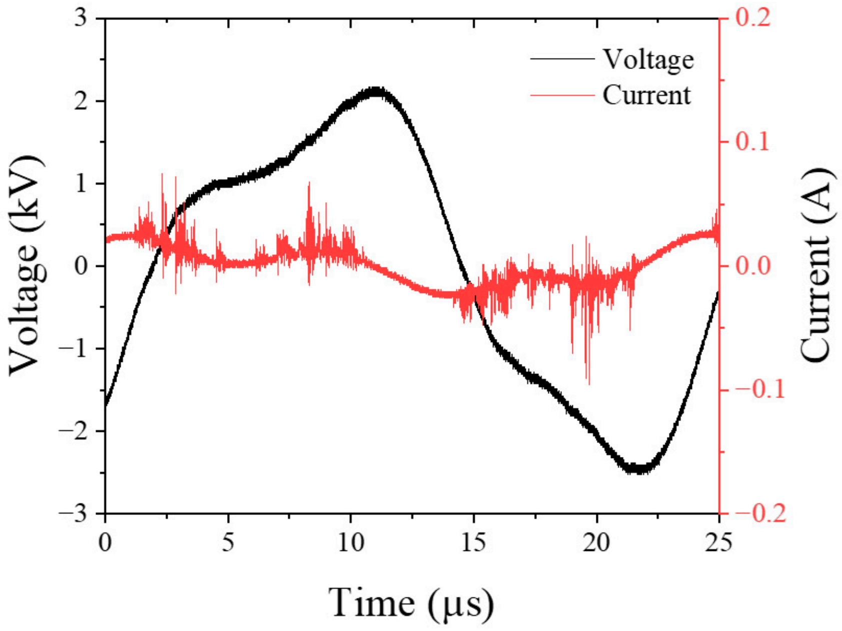

Supplementary Figure S2. Electrical characteristics of DBD, voltage (black line), and current curve (red line) versus time**.**

The chemical characteristics of DBD are illustrated in Supplementary Figure S3. These properties were assessed using Gas Fourier Transform Infrared Spectroscopy (Matrix-MG5 gas analyzer, Bruker, USA). Generally, in low-temperature air plasma below approximately 400K, O_3 and N_2O_5 are generated due to their temperature-dependent characteristics [76]. In this experiment, the transmittance of reactive oxygen and nitrogen species (RONS) has also been recorded, including O_3 (from 960 cm^{-1} to 1080 cm^{-1} and 2040 cm^{-1} to 2140 cm^{-1}), N_2O_5 (1220 cm^{-1} to 1260 cm^{-1}), and N_2O (2150 cm^{-1} to 2270 cm^{-1}). Additionally, the transmittance of H_2O (from 1350 cm^{-1} to 1860 cm^{-1}) and CO_2 (from 2300 cm^{-1} to 2400 cm^{-1}) resulting from the ambient air has been observed.

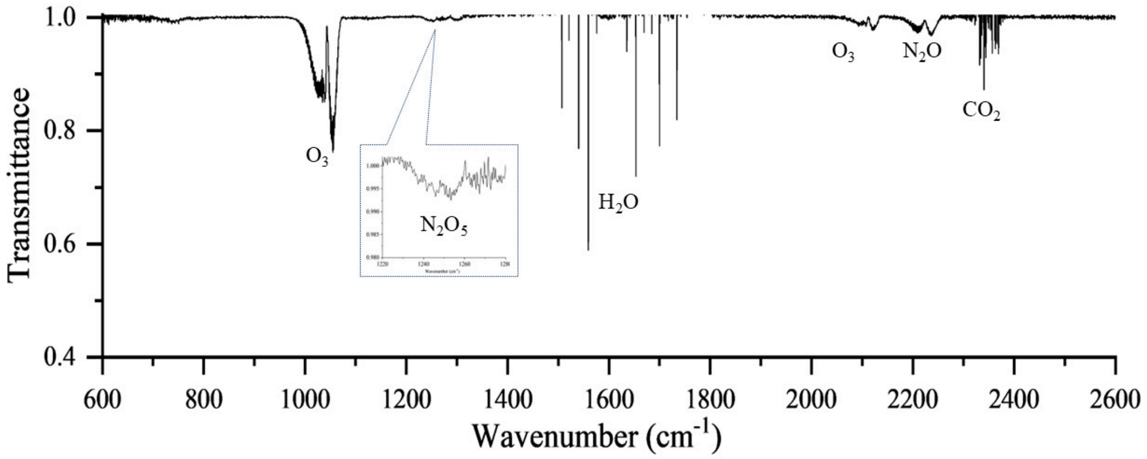

Supplementary Figure S3. The chemical characteristics of DBD by Gas Fourier Transform Infrared Spectroscopy (Gas-FTIR)

Supplementary Table S1. The antioxidant activity in shoots of 14-day-old water convolvulus plants after the seeds were treated with plasma for 0, 5, 10, and 20 min, expressed as milligram equivalents of standard per gram dry weight.

Plasma treatment time (min)	DPPH (mg TE/g DW)	ABTS (mg TE/g DW)	FRAP (mg $FeSO_4$/g DW)
0	8.4 ± 0.48[b]	7.3 ± 0.51[c]	28.6 ± 2.2[b]
5	8.1 ± 0.48[b]	10.0 ± 0.39[b]	27.6 ± 4.5[b]
10	11.2 ± 0.53[a]	13.3 ± 1.0[a]	39.1 ± 4.9[a]
20	8.6 ± 0.59[b]	6.3 ± 0.21[d]	27.7 ± 4.8[b]

* Each value represents the mean and standard deviation of replicate measurements; $n = 9$. The letters indicate a statistically significant difference across datasets, as determined by a one-way ANOVA followed by post hoc Tukey's HSD test at $p < 0.01$.
